# 48 Metformin Rescues the Aging-induced Failure of Post-burn White Adipose Browning

**DOI:** 10.1093/jbcr/irac012.051

**Published:** 2022-03-23

**Authors:** Dalia Barayan, Carly M Knuth, Marc G Jeschke

**Affiliations:** University of Toronto, Toronto, Ontario; University of Toronto, Toronto, Ontario; U of T, Toronto, Ontario

## Abstract

**Introduction:**

Severe burns are responsible for an estimated 300,000 deaths per year worldwide. While modern burn care has markedly improved survival for pediatric and adult patients, this is sadly not the case for one population: the elderly. Despite being the fastest-growing demographic in North America, burn patients over the age of 60 years have the highest mortality and morbidity rates. Recent evidence suggests that progressive aging induces several structural and functional alterations which impair the capacity of older trauma patients to adequately respond to stress. Indeed, it was discovered that reduced survival in elderly burn patients is associated with the failure to initiate the browning of white adipose tissue (WAT) —a hallmark of the systemic response to injury commonly observed in adults. Interestingly, the widely used hypoglycemic drug metformin has been found to protect against aging-induced metabolic decline in various pathological conditions. Thus, we investigated the anti-aging effects of metformin on the metabolic deterioration of post-burn WAT responses in elderly patients and mice after injury.

**Methods:**

Human WAT was obtained from elderly patients admitted to our burn center. Elderly (75-week) mice received a full-thickness scald burn and/or daily intraperitoneal injections of metformin (100 mg/kg) for 7 days. The inguinal WAT was harvested for histological analyses. Mitochondrial respiration was measured via Seahorse XF96. Gene and protein expression was assessed via RT-PCR and western blot, respectively.

**Results:**

Post-burn metformin treatment restores the thermogenic activation of WAT in elderly patients and mice, reflected by the increased expression of key browning markers, UCP-1 and PGC-1α (p< 0.05). This was accompanied by higher mitochondrial respiration, improved lipolysis (p< 0.05) and increased fat wasting (p< 0.01) relative to control counterparts. The anti-aging effects of metformin appeared to be mediated by AMPK, which consequently increased [NAD^+^] (p< 0.01), thereby promoting activation of the longevity-specific enzyme Sirt-1 (p< 0.05).

**Conclusions:**

Here, we show that post-burn metformin treatment effectively rejuvenates adaptive metabolic responses in elderly WAT by targeting key longevity pathways which rescue the age-dependent loss of being back to youthful levels. Our findings support the potential of anti-aging modalities to improve care and outcomes in elderly burned patients.

